# Real-world trends in radiotherapy modalities for esophageal cancer: analysis of a nationwide claims database in Japan

**DOI:** 10.1007/s10388-026-01248-6

**Published:** 2026-08-31

**Authors:** Kazuya Takeda, Shinsaku Okuda, Rei Umezawa, Takaya Yamamoto, Noriyoshi Takahashi, Hiroshi Onishi, Keiichi Jingu

**Affiliations:** 1 Department of Radiation Oncology, Tohoku University Graduate School of Medicine, 1-1 Seiryo-machi, Aoba-ku, Sendai, 980-8574 Miyagi Japan; 2 Department of Radiation Oncology, South Miyagi Medical Center, 38-1 Nishi, Ogawara, Shibata, 989-1253 Miyagi Japan; 3 Department of Radiology, Faculty of Medicine, University of Yamanashi, 4-4-37 Takeda, Kofu, 400-8510 Yamanashi Japan

**Keywords:** Esophageal neoplasms, Radiotherapy, Dose fractionation, Administrative claims, Practice patterns

## Abstract

**Background:**

Recently, the application of Intensity-Modulated Radiotherapy (IMRT) and changes in fractionation schedules have been increasingly introduced in radiotherapy practice for esophageal cancer. However, the extent of their adoption and prevalence in real-world clinical practice remains unclear. This study aimed to describe nationwide trends in radiotherapy for esophageal cancer using a large-scale health insurance claims database.

**Methods:**

Using the DeSC database, we identified patients newly diagnosed with esophageal cancer between April 2017 and March 2023. Patients treated with definitive radiotherapy (defined as 25–35 treatment days in patients without distant metastasis) were selected. We analyzed temporal trends in radiotherapy, regional differences, and the incidence of radiation pneumonitis.

**Results:**

Among patients with an identified initial treatment, the proportion of radiotherapy declined from 28.6% in 2017 to 19.6% in 2022. A total of 1,324 patients were included in the definitive radiotherapy group. The use of IMRT increased substantially from 10.7% in 2017 to 44.9% in 2022. Regarding fractionation, treatments of 25–28 days increased from 25.0% to 35.9%, while those of 31–35 days decreased. While CDDP/5-FU remained the dominant concurrent chemotherapy, its proportion declined as other platinum-based regimens increased. Urban facilities showed significantly higher implementation of Image-Guided Radiotherapy (IGRT) compared with rural facilities. The cumulative incidence of hospitalization for radiation pneumonitis at 12 months was 1.5%. The cumulative incidence was not significantly altered by IMRT use (sHR: 0.32, *p* = 0.13), whereas it was lower in patients receiving IGRT (sHR: 0.39, *p* = 0.04).

**Conclusions:**

This nationwide study demonstrated increased use of advanced techniques including IMRT and IGRT, and shorter fractionation schedules in radiotherapy for esophageal cancer in Japanese clinical practice.

## Introduction

Radiotherapy is a definitive treatment modality for esophageal cancer, alongside surgery and endoscopic resection. While radiotherapy was historically associated with low cure rates and late adverse events affecting the heart and lungs in long-term survivors, improvements both in oncological outcomes and safety profiles have been reported [1–5]. These improvements are attributed to advancements in radiotherapy technology that enhance target coverage while sparing normal organs, as well as the optimization of radiation dose and concurrent chemotherapy.

However, advanced treatment techniques often require sophisticated equipment and skilled staff, typically resulting in a time lag for widespread adoption [6]. While recent studies have reported on the nationwide use of advanced techniques such as intensity-modulated radiotherapy (IMRT) and hypofractionated regimens in overall radiotherapy populations, disease-specific data for esophageal cancer remain limited [7–9]. Furthermore, detailed real-world data regarding adverse events, such as radiation pneumonitis, and regional disparities in care remain limited. Such information is essential for the optimal implementation of newer treatment modalities across different regions.

The objective of this study was to describe the current status and temporal trends of radiotherapy for esophageal cancer in Japan, including the incidence of adverse events and regional and facility-level differences, by analyzing a large-scale health insurance claims database.

## Materials and methods

### Data source

This study utilized the DeSC database (DeSC Healthcare, Tokyo, Japan), a commercial claims database in Japan that captures insured medical procedures and drug prescriptions at the individual patient level. Japan has a universal health insurance system requiring all residents to enroll in public medical insurance. The DeSC database comprises data provided by multiple insurers, including the Health Insurance Society for corporate employees and their families, National Health Insurance for self-employed individuals and retirees, and the Medical Care System for the Elderly in the Latter Stage of Life, primarily for those aged 75 and older. This composition enables coverage of a wide age range, including both young and elderly patients. For this study, a dataset containing information from February 2014 to February 2025 was obtained on September 5, 2025.

### Study population

We identified patients based on diagnosis codes for esophageal cancer (ICD-10 codes: C15 and D00.1) and defined the date of the first record of these codes as the index date. Patients newly diagnosed from April 2017 to March 2023 were included in the study. We excluded patients with a database enrollment period of less than one year prior to the index date and those with diagnosis codes for other malignant neoplasms recorded in the preceding year. For eligible patients, claim codes for surgical therapy, endoscopic resection, and radiotherapy of 25 to 35 days initiated within one year of the index date were recorded. The treatment modality associated with the earliest claim code among surgery, endoscopic resection, and radiotherapy was defined as the initial treatment. Chemotherapy was classified as the initial treatment only when none of the other three modalities (surgery, endoscopic resection, or radiotherapy) were claimed. Because this study focused on definitive radiotherapy, the final analysis cohort was further restricted by excluding patients with diagnosis codes for other cancers or distant metastases in the year preceding treatment initiation.

Perioperative and salvage therapies were identified within fixed time windows around each initial treatment. For the surgery group, preoperative radiotherapy (20–24 fractions) and preoperative chemotherapy were defined as those administered between the index date and the day before surgery, while postoperative radiotherapy (20–35 fractions) and postoperative chemotherapy were defined as those between postoperative day 1 and day 180. For the endoscopic resection group, salvage surgery and salvage radiotherapy (20–35 fractions) were defined as those administered between the day after the procedure and day 180. For the radiotherapy group, neoadjuvant chemotherapy was defined as a chemotherapy prescription between the index date and 14 days before radiotherapy initiation, adjuvant chemotherapy as a prescription between 14 and 180 days after radiotherapy completion, and salvage surgery as a claim between the day after radiotherapy and day 180. In this process, post-treatment therapies could not be classified as either prophylactic or recurrence-directed. A 14-day interval between radiotherapy and chemotherapy was applied to account for the timing variability of chemotherapy prescriptions in concurrent chemoradiotherapy. The 20–35-fraction range was chosen to cover schedules from representative perioperative regimens, such as 41.4 Gy in 23 fractions [10] and its variant of 40 Gy in 20 fractions, through definitive regimens.

### Definitive radiotherapy group and variables

Among patients whose initial treatment was radiotherapy, those who received 25 to 35 fractions and had no diagnosis codes for other cancers or distant metastases within the year prior to the start of radiotherapy were defined as the definitive radiotherapy group. This range was selected to encompass the representative definitive schedules in Japan, namely 60 Gy in 30 fractions [11, 12] and 50.4 Gy in 28 fractions [13, 14], along with their variants such as 66–70 Gy in 33–35 fractions and 50 Gy in 25 fractions. The number of radiotherapy fractions was derived from the number of days with claims for radiotherapy management and irradiation fees; treatment dates separated by 14 days or less were considered part of a single continuous course. Although particle therapy and stereotactic body radiotherapy (SBRT) are typically billed as a single claim regardless of the number of fractions, these modalities were not covered by insurance for localized esophageal cancer during the study period; thus, their impact was considered negligible.

For the definitive radiotherapy group, additional detailed characteristics were extracted. Radiotherapy modality was determined based on billing codes within the treatment course, prioritized in the following order: brachytherapy, particle therapy, SBRT, intensity-modulated radiotherapy (IMRT), ≥ 4-field irradiation (including rotational or conformal irradiation), 2–3 field irradiation, and other external beam radiotherapy. Concurrent chemotherapy was defined as the prescription of chemotherapy agents during the period from the start to the end of the radiotherapy course, with regimen types categorized as CDDP/5-FU, other platinum-based, non-platinum, or none. Additionally, we assessed whether specific add-on fees were billed at least once during the course, including those for image-guided radiotherapy (IGRT) and outpatient radiotherapy. Hospitals were classified as Prefectural Designated Cancer Care Hospitals (including the two National Cancer Centers in this study) or other hospitals. Facility location was classified based on the medical institution initiating radiotherapy, categorized into urban areas (Tokyo’s 23 special wards, designated cities, and core cities) and rural areas (all other regions).

### Adverse events

To examine whether the use of IMRT or IGRT alters the risk of adverse events, we analyzed the incidence of radiation pneumonitis in the definitive radiotherapy group. We compared outcomes between IMRT and non-IMRT groups, and between IGRT and non-IGRT groups. Two endpoints were defined: (1) the first record of a radiation pneumonitis diagnosis, and (2) the first hospitalization where radiation pneumonitis was the primary diagnosis. Hospitalization events were identified using Diagnosis Procedure Combination (DPC) records with radiation pneumonitis coded as the principal diagnosis.

### Statistical analysis

Continuous variables were summarized as medians and interquartile ranges (IQR), while categorical variables were presented as frequencies and proportions. Temporal trends in treatment characteristics were summarized by Japanese fiscal year (April to March) and visualized using descriptive graphs. Differences between groups (urban and rural areas, and Prefectural Designated Cancer Care Hospitals and other hospitals) were evaluated using the Chi-square test for categorical variables and the Mann-Whitney U test for continuous variables. The cumulative incidence of radiation pneumonitis was estimated using the cumulative incidence function (CIF), with death treated as a competing risk. The Fine-Gray subdistribution hazard model was used to estimate subdistribution hazard ratios (sHR) and 95% confidence intervals (CI) for the comparison between IMRT and non-IMRT groups, and IGRT and non-IGRT groups. All statistical tests were two-sided, with significance set at *p* < 0.05.

Data extraction and processing were performed using Python version 3.1.4 (Python Software Foundation, Wilmington, DE, USA) and the DuckDB library version 1.4.1 (DuckDB Labs, Amsterdam, Netherlands). Statistical analyses were conducted using R version 4.5.1 (R Foundation for Statistical Computing, Vienna, Austria).

### Ethical considerations

This study was an observational study utilizing an anonymized database; therefore, the requirement for informed consent was waived. The study was approved by the Ethics Committee of the Tohoku University Graduate School of Medicine (Approval Number: 2025-1-452).

### Use of generative AI

Generative artificial intelligence (AI) tools, including ChatGPT, Gemini, Claude, and Cursor, were used to assist in drafting analysis code and the manuscript. The final code and manuscript were verified, revised, and finalized under the sole responsibility of the authors.

## Results

### Distribution of all cases

**Fig. 1:**
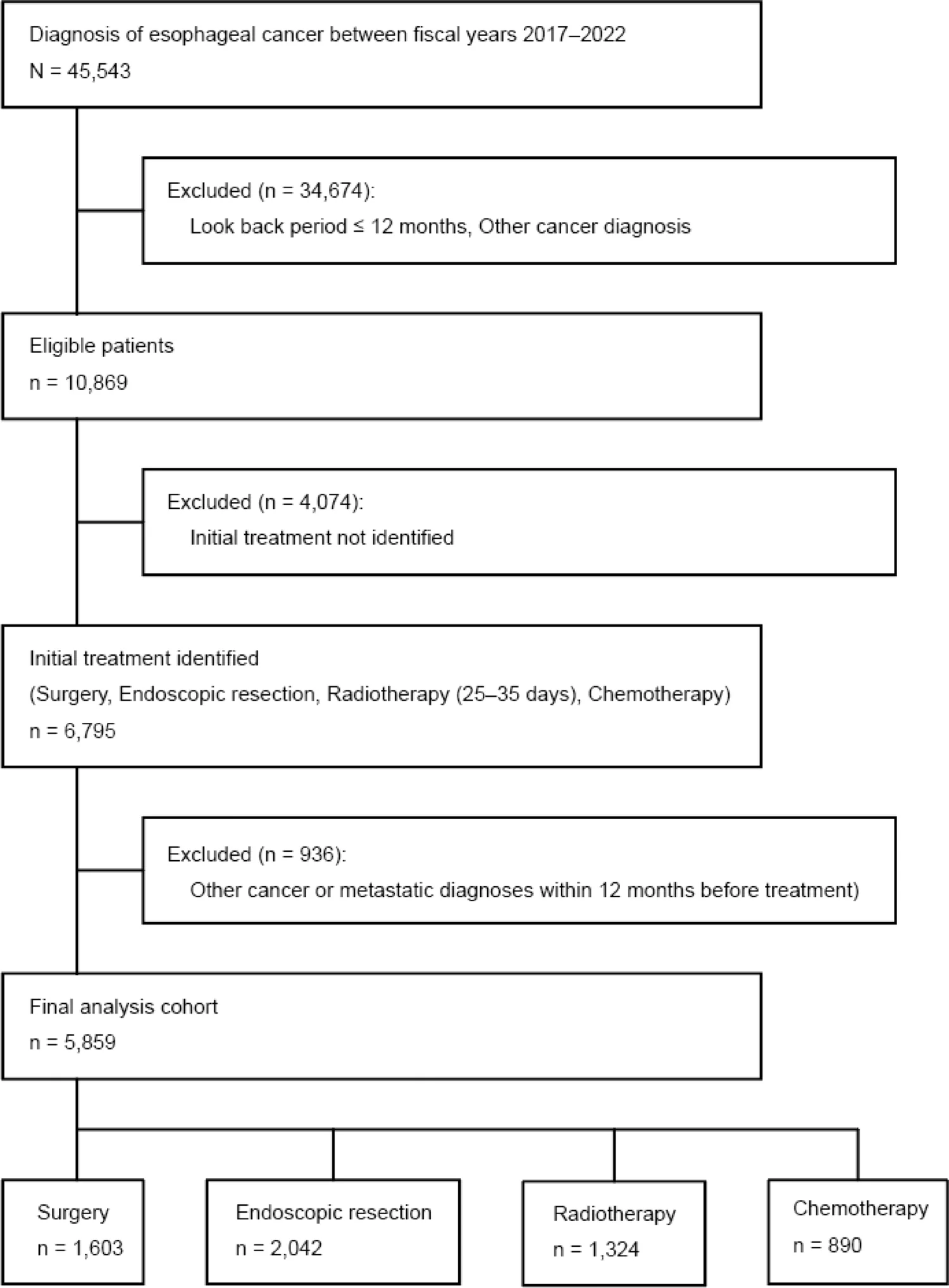
Study flow diagram

The patient selection flow and the number of patients at each stage are shown in Fig. 1. Of 45,543 patients who had an initial diagnosis of esophageal cancer between FY2017 and FY2022, 10,869 patients met eligibility criteria. Of these, 6,795 patients had an initial treatment identified within one year of diagnosis. After excluding patients with diagnosis codes for other cancers or distant metastases at the time of initial treatment, the final analysis cohort of 5,859 patients comprised surgery (*n* = 1,603), endoscopic resection (*n* = 2,042), radiotherapy (*n* = 1,324), and chemotherapy (*n* = 890).

The distribution of initial treatment and combination therapies, and their temporal changes by fiscal year are shown in Table 1. Endoscopic resection increased from 27.8% in FY2017 to 38.7% in FY2022, whereas surgery decreased from 27.4% to 22.8%, and radiotherapy decreased from 28.6% to 19.6%. Among surgery patients, neoadjuvant chemotherapy and radiotherapy were administered in 64.5% and 3.6%, respectively, while postoperative chemotherapy and radiotherapy were used in 23.7% and 4.2% within 180 days. Among endoscopic resection patients, salvage surgery and salvage radiotherapy were performed in 2.5% and 4.0%, respectively. Among definitive radiotherapy patients, neoadjuvant chemotherapy was used in 14.4%, adjuvant chemotherapy in 41.4%, and salvage surgery in 1.8%.

**Table 1:** Distribution of initial treatment by fiscal year

Category	Variable	FY2017 (*n* = 489)	FY2018 (*n* = 619)	FY2019 (*n* = 1,082)	FY2020 (*n* = 1,158)	FY2021 (*n* = 1,315)	FY2022 (*n* = 1,196)	Total (*n* = 5,859)
Surgery	Overall	134 (27.4%)	198 (32.0%)	307 (28.4%)	329 (28.4%)	362 (27.5%)	273 (22.8%)	1,603 (27.4%)
	Neoadjuvant chemotherapy	71 (53.0%)	112 (56.6%)	213 (69.4%)	213 (64.7%)	246 (68.0%)	179 (65.6%)	1,034 (64.5%)
	Neoadjuvant RT	5 (3.7%)	12 (6.1%)	7 (2.3%)	14 (4.3%)	11 (3.0%)	8 (2.9%)	57 (3.6%)
	Adjuvant chemotherapy	24 (17.9%)	46 (23.2%)	67 (21.8%)	73 (22.2%)	104 (28.7%)	66 (24.2%)	380 (23.7%)
	Adjuvant RT	3 (2.2%)	19 (9.6%)	15 (4.9%)	14 (4.3%)	9 (2.5%)	8 (2.9%)	68 (4.2%)
Endoscopic resection	Overall	136 (27.8%)	201 (32.5%)	373 (34.5%)	388 (33.5%)	481 (36.6%)	463 (38.7%)	2,042 (34.9%)
	Salvage surgery	7 (5.1%)	5 (2.5%)	11 (2.9%)	10 (2.6%)	13 (2.7%)	6 (1.3%)	52 (2.5%)
	Salvage RT	5 (3.7%)	10 (5.0%)	10 (2.7%)	15 (3.9%)	25 (5.2%)	17 (3.7%)	82 (4.0%)
Radiotherapy	Overall	140 (28.6%)	145 (23.4%)	255 (23.6%)	272 (23.5%)	278 (21.1%)	234 (19.6%)	1,324 (22.6%)
	Neoadjuvant chemotherapy	22 (15.7%)	23 (15.9%)	36 (14.1%)	34 (12.5%)	39 (14.0%)	36 (15.4%)	190 (14.4%)
	Adjuvant chemotherapy	52 (37.1%)	70 (48.3%)	95 (37.3%)	114 (41.9%)	121 (43.5%)	96 (41.0%)	548 (41.4%)
	Salvage surgery	0 (0.0%)	0 (0.0%)	6 (2.4%)	6 (2.2%)	7 (2.5%)	5 (2.1%)	24 (1.8%)
Chemotherapy	Overall	79 (16.2%)	75 (12.1%)	147 (13.6%)	169 (14.6%)	194 (14.8%)	226 (18.9%)	890 (15.2%)

Initial treatment was defined as the treatment modality associated with the earliest claim code within one year of diagnosis. Radiotherapy was identified when the treatment days was between 25 and 35. Chemotherapy was assigned as the initial treatment only in the absence of claims for surgery, endoscopic resection, or radiotherapy within one year of diagnosis. Neoadjuvant therapies were identified between the index date and the day before initial treatment, and adjuvant therapies between the day after initial treatment completion and day 180. For radiotherapy, chemotherapy within 14 days before or after the radiotherapy course was excluded as potentially concurrent chemoradiotherapy. Data are presented as n (%)

* FY* fiscal year (April to March)

### Distribution and trends in the definitive radiotherapy group

**Fig. 2:**
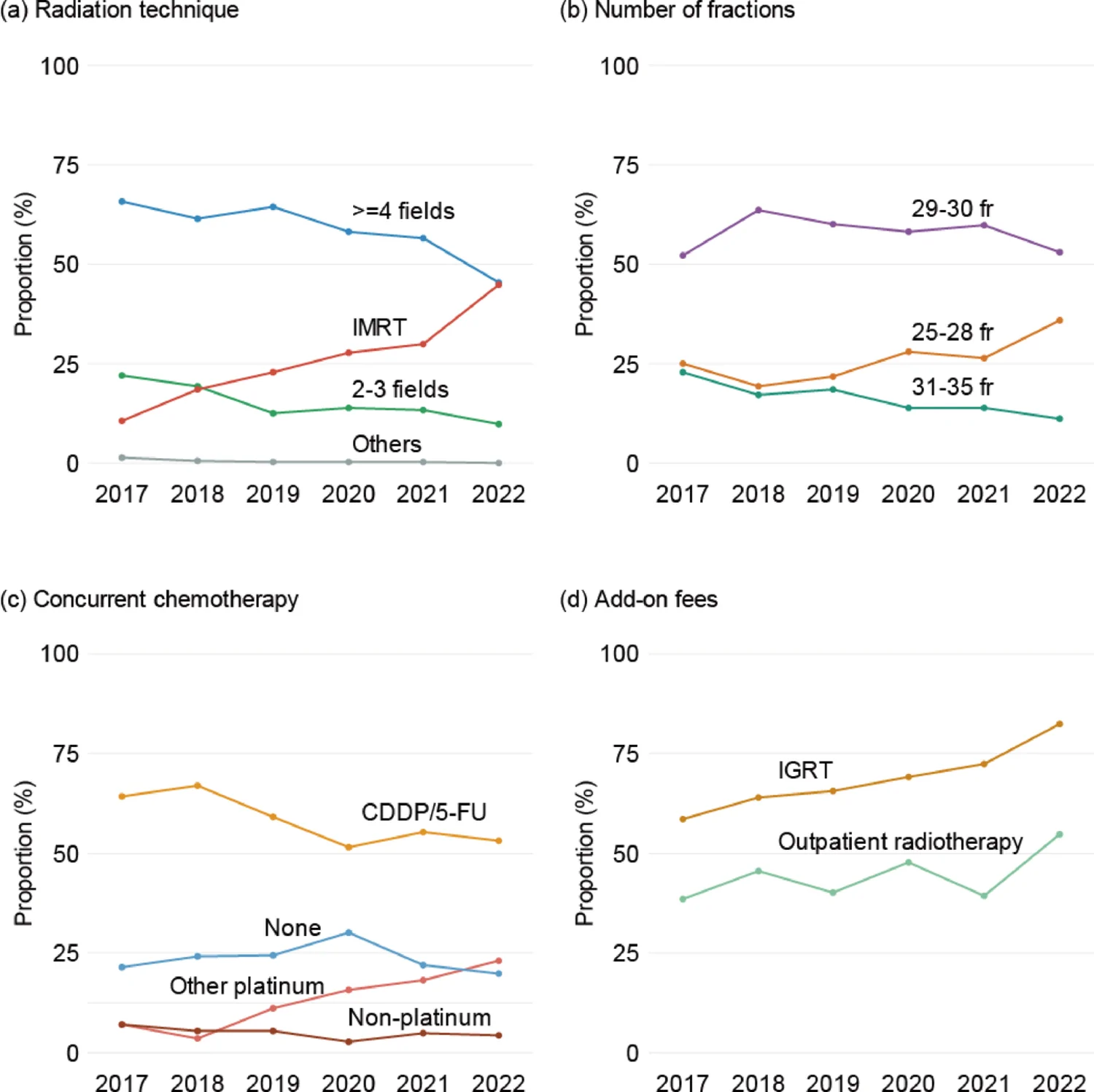
Temporal trends in treatment characteristics among patients receiving definitive radiotherapy for esophageal cancer. **a** Radiation technique: proportion of patients treated with 2–3 fields, ≥ 4 fields, IMRT, and others., **b** Number of fractions: proportion of patients receiving 25–28, 29–30, and 31–35 fractions, **c** Concurrent chemotherapy: proportion of patients receiving CDDP/5-FU, other platinum-based regimens, non-platinum regimens, or no chemotherapy, **d** Add-on fees: proportion of patients with billing for IGRT and outpatient radiotherapy fees.* IMRT* intensity-modulated radiotherapy,* CDDP* cisplatin, * 5-FU* 5-fluorouracil,* IGRT* image-guided radiotherapy

The treatment details and temporal changes for the 1,324 patients who received radiotherapy as initial treatment are shown in Table 2; Fig. 2. Regarding irradiation techniques, 2–3 field irradiation decreased from 22.1% to 9.8% and ≥ 4-field irradiation from 65.7% to 45.3%, while IMRT increased from 10.7% in FY2017 to 44.9% in FY2022. The number of treatment days was most frequently 29–30, with the proportion remaining relatively stable across years (range: 52.1–63.4%). However, the proportion of 25–28 day treatment increased from 25.0% to 35.9%, while that of 31–35 days treatment decreased from 22.9% to 11.1% during the study period.

**Table 2:** Characteristics of patients receiving definitive radiotherapy

Variable	Category	Overall (*n* = 1324)
Diagnosis year	FY2017	140 (10.6%)
	FY2018	145 (11.0%)
	FY2019	255 (19.3%)
	FY2020	272 (20.5%)
	FY2021	278 (21.0%)
	FY2022	234 (17.7%)
Sex	Female	259 (19.6%)
	Male	1065 (80.4%)
Age at diagnosis	Median [IQR]	77 [71, 81]
Prefectural designated cancer care hospital	Yes	211 (15.9%)
Facility location	Urban	707 (53.4%)
	Rural	381 (28.8%)
	Unknown	236 (17.8%)
Lymph node metastasis	Yes	209 (15.8%)
Radiation technique	2–3 fields	189 (14.3%)
	≥ 4 fields	766 (57.9%)
	IMRT	363 (27.4%)
	Others	6 (0.5%)
Number of fractions	25–28 fr	351 (26.5%)
	29–30 fr	766 (57.9%)
	31–35 fr	207 (15.6%)
IGRT additional fee	Yes	924 (69.8%)
Outpatient RT fee	Yes	589 (44.5%)
Concurrent chemotherapy	CDDP/5-FU	756 (57.1%)
	Other platinum	190 (14.4%)
	Non-platinum	62 (4.7%)
	None	316 (23.9%)

Data are presented as n (%) or median [IQR]. Definitive radiotherapy was defined as 25–35 treatment days in patients without distant metastasis

*FY* fiscal year (April to March),* IQR* interquartile range,* IMRT* intensity-modulated radiotherapy,* fr* fractions,* IGRT* image-guided radiotherapy,* RT* radiotherapy,* CDDP* cisplatin,* 5-FU* 5-fluorouracil

Concurrent chemotherapy was administered in 75.7–80.4% of cases, except for a transient decrease to 69.9% in FY2020. The most common regimen was CDDP/5-FU, but its proportion decreased from 64.3% in FY2017 to 53.0% in FY2022, while other platinum-based regimens increased from 7.1% to 23.1%. Regarding add-on fees, the IGRT billing rate rose from 58.6% to 82.5%. The outpatient radiotherapy billing rate also increased, from 38.6% to 54.7%.

### Incidence of adverse events

**Fig. 3:**
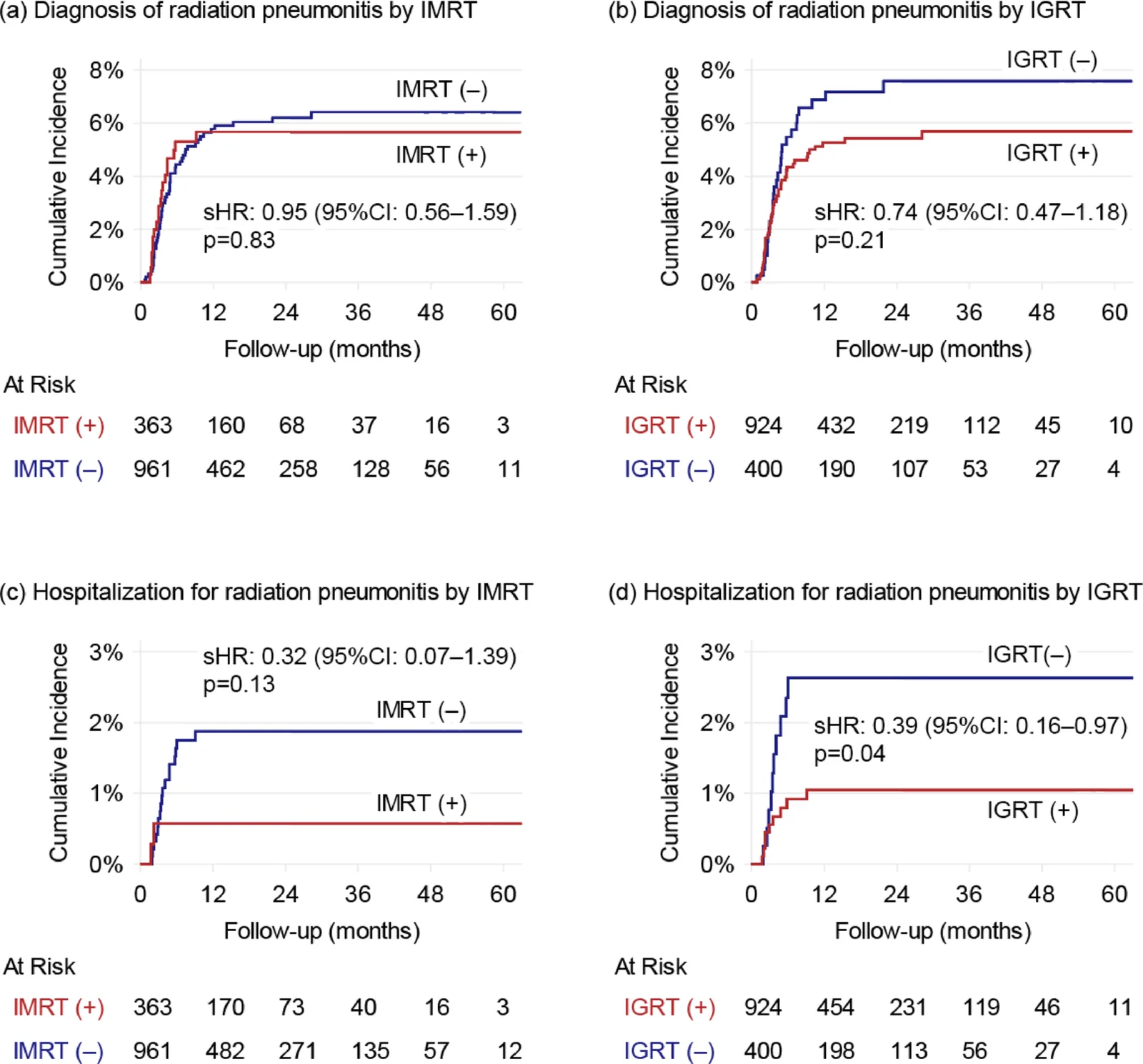
Cumulative incidence of radiation pneumonitis in patients receiving definitive radiotherapy for esophageal cancer, comparing IMRT and non-IMRT groups. Cumulative incidence plots with death as a competing risk. The first diagnosis of radiation pneumonitis by **a** IMRT use and **b** IGRT use. The first hospitalization for radiation pneumonitis by **c** IMRT use and **d** IGRT use. Subdistribution hazard ratios (sHR) and 95% confidence intervals (CI) were calculated using the Fine-Gray model. * IMRT* intensity-modulated radiotherapy, * IGRT* image-guided radiotherapy

In the definitive radiotherapy group, the cumulative incidence of the first record of radiation pneumonitis diagnosis at 12 and 24 months was 5.8% and 6.1%, respectively. The sHR for the IMRT group compared with the non-IMRT group was 0.95 (95% CI 0.56–1.59, *p* = 0.83; Fig. 3a), and that for the IGRT group compared with the non-IGRT group was 0.74 (95% CI 0.47–1.18, *p* = 0.21; Fig. 3b).

The cumulative incidence of the first hospitalization for radiation pneumonitis was 1.5% at both 12 and 24 months. The sHR for the IMRT group was 0.32 (95% CI 0.07–1.39, *p* = 0.13; Fig. 3c) and that for the IGRT group was 0.39 (95% CI 0.16–0.97, *p* = 0.04; Fig. 3d).

### Differences in definitive radiotherapy by region and facility category

Facility location information was available for 1,088 patients (82.2%) in the definitive radiotherapy group, of whom 707 (65.0%) were treated at urban facilities (Table 3). IMRT use was higher in urban areas (30.0%) than in rural areas (24.9%), although the difference was not statistically significant (*p* = 0.07). The distribution of treatment days differed between urban and rural areas, with a higher proportion of 25–28 day and a lower proportion of 29–30 day treatments in urban areas (*p* = 0.049). The distribution of concurrent chemotherapy regimens also differed significantly between urban and rural areas (*p* = 0.004), with a higher overall use of any chemotherapy in urban areas (78.6% vs. 71.9%) and a higher proportion of platinum-based regimens other than CDDP/5-FU. Add-on fees for IGRT and for outpatient radiotherapy were significantly higher in urban areas (*p* < 0.001).

**Table 3:** Comparison of treatment characteristics between urban and rural facilities

Variable	Category	Urban (*n* = 707)	Rural (*n* = 381)	*P*-value
Sex	Female	142 (20.1%)	76 (19.9%)	1.00
	Male	565 (79.9%)	305 (80.1%)	
Age at diagnosis	Median [IQR]	77 [71, 81]	77 [70, 81]	0.60
Lymph node metastasis	Yes	118 (16.7%)	59 (15.5%)	0.67
Radiation technique	2–3 fields	84 (11.9%)	60 (15.7%)	0.07
	≥ 4 fields	411 (58.1%)	225 (59.1%)	
	IMRT	212 (30.0%)	95 (24.9%)	
	Others	0 (0.0%)	1 (0.3%)	
Number of fractions	25–28 fr	211 (29.8%)	88 (23.1%)	0.049
	29–30 fr	390 (55.2%)	225 (59.1%)	
	31–35 fr	106 (15.0%)	68 (17.8%)	
IGRT additional fee	Yes	528 (74.7%)	232 (60.9%)	< 0.001
Outpatient radiation fee	Yes	355 (50.2%)	143 (37.5%)	< 0.001
Concurrent chemotherapy	CDDP/5-FU	411 (58.1%)	216 (56.7%)	0.004
	Other platinum	113 (16.0%)	36 (9.4%)	
	Non-platinum	32 (4.5%)	22 (5.8%)	
	None	151 (21.4%)	107 (28.1%)	

Data are presented as n (%) or median [IQR]. Urban areas were defined as Tokyo’s 23 special wards, designated cities, and core cities. P-values were calculated using the Chi-square test for categorical variables and the Mann-Whitney U test for continuous variables

*IQR* interquartile range,* IMRT* intensity-modulated radiotherapy,* fr* fractions, *IGRT* image-guided radiotherapy,* RT* radiotherapy,* CDDP* cisplatin,* 5-FU* 5-fluorouracil

Of all 1,324 radiotherapy cases, 211 (15.9%) were treated at Prefectural Designated Cancer Care Hospitals (Table 4). In the designated hospitals, the use of IMRT, 29–30 day treatments, IGRT, and concurrent chemotherapy was more frequent than in other hospitals (*p* < 0.001 for each).

**Table 4:** Comparison of treatment characteristics between prefectural designated cancer care hospitals and other hospitals

Variable	Category	Prefectural designated cancer care hospital (*n* = 211)	Other hospitals (*n* = 1,113)	*P*-value
Sex	Female	40 (19.0%)	219 (19.7%)	0.88
	Male	171 (81.0%)	894 (80.3%)	
Age at diagnosis	Median [IQR]	75 [68, 79]	77 [71, 81]	< 0.001
Lymph node metastasis	Yes	34 (16.1%)	175 (15.7%)	0.97
Radiation technique	2–3 fields	12 (5.7%)	177 (15.9%)	< 0.001
	≥ 4 fields	116 (55.0%)	650 (58.4%)	
	IMRT	82 (38.9%)	281 (25.2%)	
	Others	1 (0.5%)	5 (0.4%)	
Number of fractions	25–28 fr	45 (21.3%)	306 (27.5%)	< 0.001
	29–30 fr	150 (71.1%)	616 (55.3%)	
	31–35 fr	16 (7.6%)	191 (17.2%)	
IGRT additional fee	Yes	178 (84.4%)	746 (67.0%)	< 0.001
Outpatient radiation fee	Yes	103 (48.8%)	486 (43.7%)	0.19
Concurrent chemotherapy	CDDP/5-FU	129 (61.1%)	627 (56.3%)	< 0.001
	Other platinum	45 (21.3%)	145 (13.0%)	
	Non-platinum	4 (1.9%)	58 (5.2%)	
	None	33 (15.6%)	283 (25.4%)	

Data are presented as n (%) or median [IQR]. P-values were calculated using the Chi-square test for categorical variables and the Mann-Whitney U test for continuous variables

*IQR* interquartile range,* IMRT* intensity-modulated radiotherapy,* fr* fractions, *IGRT* image-guided radiotherapy,* RT* radiotherapy,* CDDP* cisplatin,* 5-FU* 5-fluorouracil

## Discussion

This study described the current status and temporal trends of radiotherapy for esophageal cancer in Japan using a large-scale insurance claims database. Previous reports using NDB Open Data have described nationwide trends in radiotherapy utilization [7–9]; however, NDB Open Data does not allow tracking of individual patient histories, precluding detailed examination of specific diseases, treatment details, and concurrent medications. To our knowledge, this is the first study in the field of radiotherapy in Japan to link diagnoses, treatment details, and post-treatment outcomes by following individual patient claims. Despite the short observation period of six years (FY2017–FY2022), the results showed recent changes in treatment practice.

Regarding initial treatment, the proportion of surgery was 27.4%. Even after adding the 76 patients (1.3%) who underwent salvage surgery following endoscopic resection or radiotherapy, the total proportion of surgery cases was 28.7%. This was lower than that reported in a previous registry analysis from Japan, in which 44.7% of patients received surgery as the primary treatment among 9,593 newly diagnosed esophageal cancer cases in 2016 [15]. The previous study was based on a registry with contributions from relatively large facilities, whereas the present study was insurance-based and included non-specialized hospitals and clinics, which may explain this difference. During the observation period, the proportion of endoscopic resection increased while that of surgery and radiotherapy decreased. The increase in endoscopic resection aligns with the JCOG0508 trial, which demonstrated the efficacy and safety of selective additional chemoradiotherapy for pathologically high-risk cases after endoscopic resection of cStage I esophageal squamous cell carcinoma [16, 17]. This strategy is currently recommended as a minimally invasive standard treatment in the Japan Esophageal Society guidelines [18]. The availability of salvage or adjuvant chemoradiotherapy as a backup option for early-stage cases likely facilitated the selection of endoscopic resection over surgery or upfront radiotherapy as initial therapy. Regarding combination therapy, neoadjuvant and adjuvant chemotherapy for surgical patients were the most commonly used combinations, likely reflecting recent evidence on perioperative pharmacotherapy [19, 20]. Salvage surgery after radiotherapy, and radiotherapy after surgery or endoscopic resection, were also performed in small proportions, consistent with recent evidence-based practice [16, 21].

Regarding treatment duration, 29–30 days was the most common, suggesting that the 60 Gy in 30 fractions regimen historically used in Japan remains prevalent [12, 22]. In contrast, major Western clinical trials have utilized 50–50.4 Gy in 25–28 fractions as the standard for definitive chemoradiotherapy [1, 2, 13, 23]. Notably, INT 0123, a phase III randomized controlled trial, failed to demonstrate a clinical benefit with dose escalation beyond 50.4 Gy [13]. Although modern techniques such as IMRT have enabled dose escalation with improved normal organ sparing, a phase III trial comparing 60 Gy with 50 Gy found no survival benefit but reported increased rates of severe pneumonitis [4, 24]. In the present study, the proportion of 31–35 day treatments decreased while that of 25–28 day treatments increased, suggesting a potential shift from high-dose regimens toward 50–50.4 Gy equivalents in Japan. However, this study used treatment days as a surrogate for dose and fractionation, and validation with dosimetric data is warranted.

In addition to changes in fractionation, the proportion of CDDP/5-FU as concurrent chemotherapy decreased while other platinum-based regimens increased over the study period. Although CDDP/5-FU has long been the standard regimen for definitive chemoradiotherapy, FOLFOX combined with radiotherapy was subsequently shown to be effective [23]. In 2019, this regimen became reimbursable under public health insurance in Japan, and the Japan Esophageal Society included it among the treatment options in a statement. The observed shift in concurrent chemotherapy patterns likely reflects this background.

Regarding treatment modalities, the proportion of conventional multi-field techniques (2–3 fields and ≥ 4 fields) declined over time, while IMRT use steadily increased. Historically, pivotal trials that established the evidence for chemoradiotherapy (e.g., RTOG 85 − 01, INT 0123) utilized 2D or early 3D treatment planning, typically delivering the initial dose via anterior-posterior opposed fields followed by an oblique field boost. However, these techniques delivered relatively high doses to the heart, and with increasing numbers of long-term survivors, late cardiopulmonary toxicity and cardiac imaging abnormalities have been reported [25–28]. Cardiac dose has been associated with pericardial effusion, cardiac events, and survival outcomes [29, 30], and several studies have demonstrated that IMRT can reduce doses to the heart and coronary arteries [31, 32]. The increase in IMRT use observed in the present study likely reflects the growing emphasis on reducing doses to normal tissue. The concurrent increase in IGRT use suggests that high-precision treatment delivery is becoming integrated into clinical practice alongside IMRT adoption.

The cumulative incidence of radiation pneumonitis at 12 months in our study was 5.8% (diagnosis-based) and 1.5% (hospitalization-based), broadly consistent with rates of Grade 2 (5–10%) and Grade 3 (1–3%) pneumonitis reported in recent chemoradiotherapy series using modern techniques [33–37]. Claims-based assessment of adverse events may over- or underestimate true incidence and cannot be directly aligned with standardized grading systems such as CTCAE; nevertheless, our findings appear reasonable. We additionally performed exploratory analyses to investigate the effect of advanced radiotherapy techniques on pneumonitis. In the thoracic region, IMRT can reduce high-dose exposure to the lungs, including V20 and mean lung dose, which are associated with severe pneumonitis, compared with conventional 2D or 3D techniques. A potential concern with IMRT is increased low-dose exposure (e.g., V5, V10), which has also been associated with pneumonitis risk [38, 39]. However, a secondary analysis of RTOG 0617 in locally advanced lung cancer found that V20, rather than V5, was associated with severe pulmonary toxicity, and reported fewer pneumonitis events with IMRT than with 3D-CRT [40]. In esophageal cancer, several studies have reported that IMRT offers dosimetric advantages, including reduced lung V20, without increasing clinical pneumonitis rates compared with 3D-CRT [32, 35]. In the present study, IMRT use did not increase the diagnosis-based incidence of pneumonitis and showed a non-significant trend toward fewer hospitalizations, consistent with previous reports. Notably, IGRT use was associated with significantly fewer pneumonitis-related hospitalizations. Existing evidence on the impact of IGRT itself remains limited; Kuo et al. reported no significant difference in pneumonitis incidence between patients with and without IGRT after propensity-score matching [41]. Because we could not adjust for detailed radiotherapy parameters such as margin size or DVH metrics, the IGRT analysis was performed as an exploratory univariate analysis. IGRT use may serve as a surrogate for several factors, including reduced lung dose via smaller margins and more advanced facility characteristics such as IMRT adoption and reduced prophylactic field volumes, which together may explain the lower pneumonitis incidence. Further validation studies comparing clinical data with claims data are needed in these contexts.

In the urban-rural stratified analysis, urban facilities tended to use IMRT in a higher proportion of the patients and showed significantly higher IGRT use. This finding is consistent with previous reports indicating that advanced treatments are more readily adopted in urban areas [6, 8]. The higher rate of concurrent chemotherapy in urban areas may reflect differences in patient characteristics, such as disease stage. The higher rate of outpatient radiotherapy fees in urban areas likely reflects the tendency for rural patients to be hospitalized throughout the treatment course due to limited access to facilities. Stratified analysis by hospital type further showed that Prefectural Designated Cancer Care Hospitals more frequently used advanced techniques (IMRT, IGRT) and concurrent chemotherapy than other hospitals. Together, these findings indicate persistent inter-facility disparities in treatment patterns and adoption of advanced treatment techniques in Japan.

This study has several limitations. First, the analysis was based on a single commercial claims database, which may not fully represent the Japanese population in terms of regional and age distribution. Second, because this analysis was based on claims data, both over- and underestimation of diagnoses and treatments are possible. The validity of our cutoffs for treatment days, course intervals, and time windows for identifying adjuvant therapies was not externally validated. Validation studies for claims-based assessment in radiotherapy remain limited, with few reports even from outside Japan [42]; further validation work using Japanese claims databases is warranted. Third, proton therapy, which has gained attention for reducing cardiopulmonary toxicity, was largely not captured. This is because most proton therapy for esophageal cancer in Japan is provided as Advanced Medical Care, which is not fully covered by public insurance and therefore not recorded in claims databases. However, the number of patients receiving particle therapy outside the public insurance system is small relative to the total, suggesting minimal impact on the overall findings. Fourth, we could not adjust for potential confounders such as disease stage, irradiated volume, or lung dose in the comparison between IMRT and non-IMRT, and between IGRT and non-IGRT groups; therefore, unmeasured confounding cannot be ruled out. Interpretation of these findings should be complemented by studies using clinical data.

## Conclusion

This study described the current status and temporal trends of radiotherapy for esophageal cancer in Japan using a large-scale claims database. We observed a trend toward shorter fractionation schedules and increases in IMRT use and in the use of platinum-based regimens other than CDDP/5-FU. The expansion of IMRT was not accompanied by an increase in radiation pneumonitis, and IGRT use appeared to be associated with fewer pneumonitis-related hospitalizations. Regional and facility-level analyses revealed higher use of IGRT and outpatient radiotherapy in urban facilities and in Prefectural Designated Cancer Care Hospitals. Further studies are warranted to establish the clinical benefits and optimal implementation of these evolving treatment approaches.

## Data Availability

The data that support the findings of this study were obtained from DeSC Healthcare, Inc. (Tokyo, Japan). These data are not publicly available due to licensing restrictions.
